# Mild therapeutic hypothermia alters neuron specific enolase as an outcome predictor after resuscitation: 97 prospective hypothermia patients compared to 133 historical non-hypothermia patients

**DOI:** 10.1186/cc8975

**Published:** 2010-04-19

**Authors:** Ingo G Steffen, Dietrich Hasper, Christoph J Ploner, Joerg C Schefold, Ekkehart Dietz, Frank Martens, Jens Nee, Anne Krueger, Achim Jörres, Christian Storm

**Affiliations:** 1 Department of Nephrology and Medical Intensive Care Medicine, Charité Universitätsmedizin Berlin, Campus Virchow-Klinikum, Augustenburgerplatz 1, 13353 Berlin, Germany; 2 Department of Biometry and Clinical Epidemiology, Charité Universitätsmedizin Berlin, Campus Mitte, Charitéplatz 1, 10117 Berlin, Germany; 3 Department of Neurology, Charité Universitätsmedizin Berlin, Campus Virchow-Klinikum, Augustenburgerplatz 1, 13353 Berlin, Germany

## Abstract

**Introduction:**

Neuron specific enolase (NSE) has been proven effective in predicting neurological outcome after cardiac arrest with a current cut off recommendation of 33 μg/l. However, most of the corresponding studies were conducted before the introduction of mild therapeutic hypothermia (MTH). Therefore we conducted a study investigating the association between NSE and neurological outcome in patients treated with MTH

**Methods:**

In this prospective observational cohort study the data of patients after cardiac arrest receiving MTH (n = 97) were consecutively collected and compared with a retrospective non-hypothermia (NH) group (n = 133). Serum NSE was measured 72 hours after admission to ICU. Neurological outcome was classified according to the Pittsburgh cerebral performance category (CPC 1 to 5) at ICU discharge.

**Results:**

NSE serum levels were significantly lower under MTH compared to NH in univariate analysis. However, in a linear regression model NSE was affected significantly by time to return of spontaneous circulation (ROSC) and ventricular fibrillation rhythm but not by MTH. The model for neurological outcome identified NSE, NSE*MTH (interaction) as well as time to ROSC as significant predictors. Receiver Operating Characteristic (ROC) analysis revealed a higher cutoff value for unfavourable outcome (CPC 3 to 5) with a specificity of 100% in the hypothermia group (78.9 μg/l) compared to the NH group (26.9 μg/l).

**Conclusions:**

Recommended cutoff levels for NSE 72 hours after ROSC do not reliably predict poor neurological outcome in cardiac arrest patients treated with MTH. Prospective multicentre trials are required to re-evaluate NSE cutoff values for the prediction of neurological outcome in patients treated with MTH.

## Introduction

Early prediction of neurological outcome in patients surviving cardiac arrest is a challenging problem. A combined approach using clinical assessment, electrophysiological studies and biochemical markers has been proven reliable in predicting poor outcome and is currently used in most centres [1-3].

Neuron specific enolase (NSE) is a gamma isomer of enolase that is located in neurons and neuroectodermal cells. Several studies have evaluated the significance of NSE to predict neurological outcome in patients after cardiac arrest. However, the results are not unanimous regarding outcome prediction and the best cutoff value for NSE [3-5]. Most authors agree that the NSE serum level after 72 hours carries the highest predictive value for neurological outcome after resuscitation [3,5]. In the large Prognosis in Postanoxic Coma Study Group (PROPAC) all patients with NSE levels >33 μg/l at any time had an unfavourable outcome [6].

With the introduction of mild therapeutic hypothermia (MTH) the treatment of patients after cardiac arrest has changed significantly in the last years. Only a minority of prognostication studies have included patients who underwent MTH after cardiac arrest. The guidelines published in 2006 by the American Academy of Neurology (AAN) on outcome prediction in comatose survivors after cardiac arrest recommend a cutoff of 33 μg/l, but were also mainly based on studies in patients not treated with hypothermia. Thus, there is an urgent need to re-evaluate the validity of previously established prognostic markers in survivors undergoing therapeutic hypothermia [7]. Therefore we conducted an observational study comparing NSE levels in patients treated with MTH with historical non-hypothermia (NH) patients.

## Materials and methods

The study protocol was approved by the local ethics committee on human research and is conducted in accordance with the guidelines of the Declaration of Helsinki. All data were collected within the normal daily intensive care routine in an anonymous fashion. The institutional review board therefore waived the need for informed patient consent. A total number of 230 patients was analyzed. The hypothermia group (n = 97) was generated between December 2006 and August 2008 from patients admitted to our medical intensive care unit (MICU) after cardiac arrest. All patients received MTH irrespective of the initial rhythm. A historical NH group in the era prior to hypothermia treatment was identified in a cohort of 133 patients admitted to our MICU between 2002 and 2004 after cardiac arrest. Detailed characteristics for all patients included in the study are given in Table 1. Therapeutic hypothermia was initiated after admission with an intravenous infusion of cold saline (4°C, 1,000 to 1,500 ml bolus) followed by surface cooling with commercially available non-invasive devices (ArcticSun2000^® ^Medivance, Louisville, Colorado, USA). The target temperature was maintained for 24 hours. Intravenous sedation and analgesia was induced in all patients by a combination of midazolam (0.125 mg/kg/h) and fentanyl (0.002 mg/kg/h) with dose adjustment as needed. Patients undergoing hypothermia received muscle relaxation with repetitive administration of pancuronium (0.1 mg/kg) in order to prevent shivering. Apart from therapeutic hypothermia, there was no further difference in critical care treatment between the two groups. In all patient's post-resuscitation treatment was conducted by physicians highly experienced in critical care according to standard operating procedures. NSE serum levels were measured 72 hours after admission to MICU with an enzyme immunoassay (Elecsys 2010, Roche Diagnostics GmbH, Mannheim, Germany). The identical test and the same laboratory were used in both groups compared in this study. Potential neurological outcome was assessed on the third day after admission to MICU by clinical examination, NSE serum levels and somato-sensory evoked potentials, when needed. In all patients, the decision to continue or discontinue treatment was taken considering the results of these tests and with the advice of an external neurologist.

**Table 1:** Baseline characteristics and neurological outcome of patients treated with mild therapeutic hypothermia (MTH) and non-hypothermia (NH) group

	NH	MTH	*P*-value
	(n = 133)	(n = 97)	
Baseline parameters			
Age (years)	64.3 (52.9 to 73.0)	60.3 (51.5 to 70.0)	0.123
Gender			
female	33 (24.8)	21 (21.6)	0.638
male	100 (75.2)	76 (78.4)	
APACHE II score	26.0 (20.0 to 32.0)	31.0 (24.0 to 34.0)	0.003
Cardiac arrest			
Out-of-hospital	101 (75.9)	81 (83.5)	0.190
In-hospital	32 (24)	16 (16.5)	
Cardiac rhythm			
Shockable rhythm	73 (54.9)	65 (67.0)	0.077
Non-shockable rhythm	60 (45.1)	32 (33.0)	
Cause of cardiac arrest			
AMI	82 (61.7)	55 (56.7)	0.688
Primary arrhythmia	24 (18)	22 (22.7)	
Respiratory	22 (16.5)	18 (18.6)	
Other	5 (3.8)	2 (2.1)	
Bystander CPR			
yes	24 (18.0)	38 (39.2)	<0.001
No	109 (82.0)	59 (60.8)	
Time to ROSC (minutes)	22 (14 to 30)	20 (12 to 27)	0.176
Total epinephrine dose (mg)	3.0 (2.0 to 5.0)	3.0 (1.0 to 4.5)	0.039
Length of ICU stay (days)	15 (7 to 26)	13 (7 to 23)	0.693
Neurological Outcome			
CPC at ICU discharge			
1 - good recovery	16 (12)	33 (34)	<0.001
2 - moderate disability	14 (10.5)	20 (20.6)	
3 - severe disability	5 (3.8)	5 (5.2)	
4 - vegetative state	34 (25.6)	5 (5.2)	
5 - death	64 (48.1)	34 (35.1)	

Data are presented as median (25th to 75th percentiles) or as absolute numbers (%). CPR, cardiopulmonary resuscitation, ROSC, return of spontaneous circulation, CPC, cerebral performance category, NSE, neuron specific enolase, AMI, acute myocardial infarction, APACHE, Acute Physiology and Chronic Health Evaluation

Clinical outcome was assessed at the time of discharge from ICU according to the Pittsburgh cerebral performance category (CPC) [8]. CPC 1 to 2 were classified as a favorable neurological outcome whereas CPC 3 to 5 were regarded as an unfavorable outcome.

The STATA software (Version 10.0; StataCorp; College Station, Texas, USA) and R (Version 2.8.1; The R Foundation for Statistical Computing, Vienna, Austria) were used for statistical analysis. According to non parametric distribution, descriptive parameters are given as median and inter-quartile range (IQR). Univariate analysis of differences between hypothermia patients and the non-hypothermia group was performed by using the Mann-Whitney-U test for non-parametric unpaired data and Fisher's exact test for dichotomous variables. Linear regression was used to analyze the association of NSE and several independent variables. Logistic regression was performed to predict neurological outcome. The association of NSE and hypothermia treatment was analyzed by adding an interaction term into the logistic regression model. Akaike information criterion (AIC) was used to select the final model. ROC analysis was performed to evaluate the value of NSE for prediction of neurological outcome. Survival data were analyzed by the Kaplan-Meier method and comparison between groups was performed by using the log-rank test.

## Results

### Baseline parameters

Results of univariate analysis of all patients (n = 230) are given in Table 1. Concerning APACHE II (Acute Physiology and Chronic Health Evaluation II) (*P *= 0.003), bystander cardiopulmonary resuscitation (CPR, *P *< 0.001) and epinephrine dosages (*P *= 0.039), significant differences were observed between patients in the hypothermia group and the non-hypothermia group. No significant differences were found for the other baseline parameters.

### NSE serum levels

**Figure 1:**
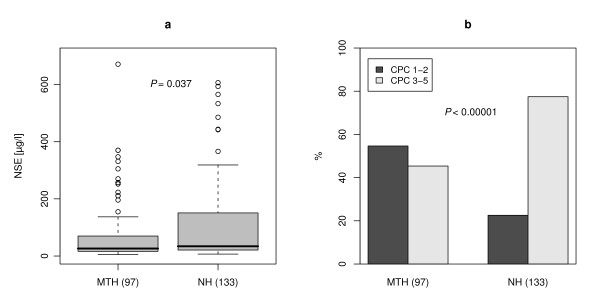
**NSE serum levels (a) and neurological outcome (b) of patients treated with mild hypothermia and non-hypothermia group**.

NSE serum levels for all patients were significantly lower under MTH treatment (median, 26.1 μg/l; IQR, 16.4 to 69.8 μg/l) compared to NH (median, 34.1 μg/l; IQR, 21.0 to 150.8 μg/l, *P *= 0.037, Figure 1a). However, in a linear regression model with adjustment for covariates, NSE was not significantly associated with mild hypothermia treatment (*P *= 0.247) whereas time to return of spontaneous circulation (ROSC) (*P *= 0.008) and ventricular fibrillation (*P *= 0.001) showed a significant effect (Table 2).

**Table 2:** Linear regression model of NSE serum levels

	Coefficient	95%-CI		*P*-value
		lower	upper	
(Intercept)	182.76	73.18	292.34	0.001
Gender female	-5.79	-42.37	30.79	0.755
Age (year)	-1.16	-2.25	-0.07	0.037
APACHE II score	0.98	-1.07	3.04	0.348
Hypothermia treatment	-16.29	-48.86	16.28	0.325
Bystander CPR	-36.89	-72.43	-1.35	0.042
Time to ROSC (min)	1.85	0.66	3.03	0.002
Ventricular fibrillation	-49.14	-80.76	-17.51	0.002

**R-squared: 0.136**				

CPR, cardiopulmonary resuscitation; ROSC, return of spontaneous circulation; CI, confidence interval; min, minutes

### Neurological outcome (CPC) and NSE ranges

**Figure 2:**
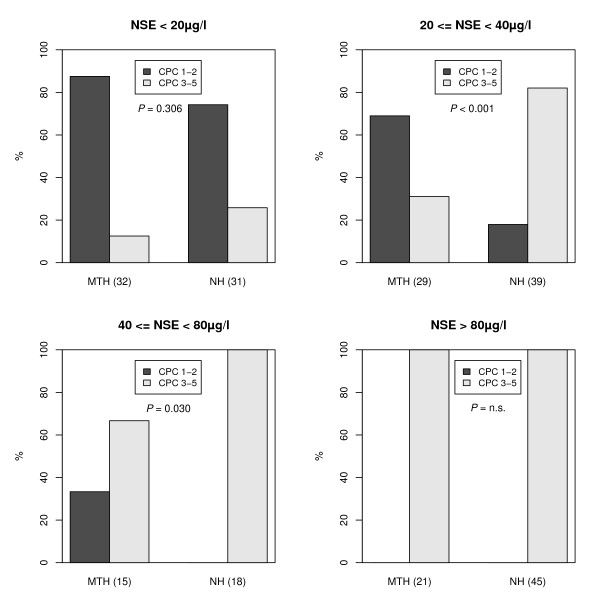
**Association of neurological outcome (CPC 1-2 vs. CPC 3-5) and NSE serum level ranges**.

Univariate analysis of neurological outcome (CPC 1 to 2 vs. CPC 3 to 5) showed a significant higher proportion (*P *< 0.001) of favorable outcome in patients treated with hypothermia compared to NH group (hypothermia 54.6% vs. control 22.5%; Figure 1b). Furthermore, the association of neurological outcome, hypothermia treatment and different ranges of NSE levels was analyzed at Day 3 (Figure 2). In patients with NSE serum levels lower than 20 μg/l the proportion of CPC 1 to 2 was higher in the hypothermia group (96.6%) versus non-hypothermia group (76.7%) without reaching significance (*P *= 0.306). A significant higher proportion of patients with good neurological outcome (CPC 1 to 2) was observed for NSE levels in the range of 20 to 40 μg/l (*P *< 0.001) as well as in the group of patients with NSE levels in the range of 40 to 80 μg/l (*P *= 0.030). Patients with an NSE greater than 80 μg/l showed a bad neurological outcome in both groups (CPC 3 to 5; MTH n = 21, NH n = 45, Figure 2).

### Interaction of CPC, NSE and hypothermia

**Figure 3:**
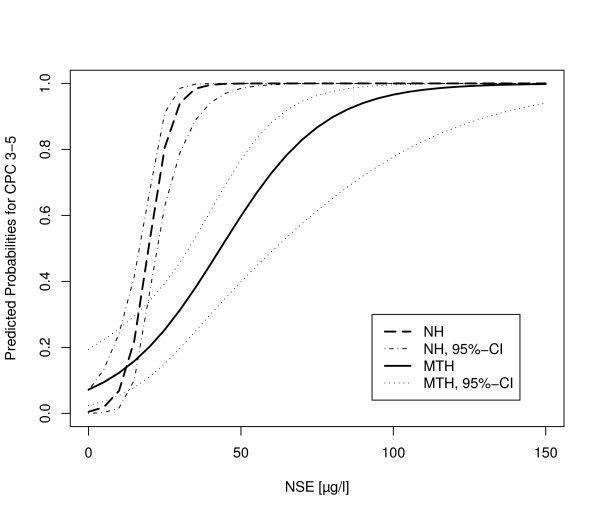
**Interaction of NSE serum level and mild hypothermia treatment (MTH) for prediction of neurological outcome**.

The association of neurological outcome (CPC 1 to 2 vs. 3 to 5), NSE serum levels and MTH was adjusted for confounders using a logistic regression model including gender, age, APACHE II-score, epinephrine dosage, bystander CPR, time to ROSC, location of cardiac arrest and initial heart rhythm as independent variables. The model for neurological outcome (CPC 1 to 2 vs. 3 to 5) identified NSE (*P *< 0.001), the interaction of NSE serum level and mild hypothermia treatment (*P *= 0.002) as well as time to ROSC (*P *= 0.017) as predictors of neurological outcome (Table 3). The interaction between NSE serum levels, applied treatment and neurological outcome is demonstrated in Figure 3 showing the predicted probabilities for CPC 3 to 5 in relation to NSE serum levels and hypothermia treatment. In the range of NSE serum levels between approximately 25 and 100 μg/l hypothermia treatment is associated with a lower probability of unfavorable neurological outcome (CPC 3 to 5) compared to the NH group whereas in the range of NSE levels >100 μg/l the probabilities for CPC 3 to 5 of hypothermia and non-hypothermia group show close approximation.

**Table 3:** Logistic regression of neurological outcome (CPC 1 to 2 versus CPC 3 to 5)

	OR	95%-CI		*P*-value
		lower	Upper	
NSE	1.31	1.15	1.50	<0.001
Hypothermia	17.44	1.03	296.62	0.048
NSE*hypothermia	0.81	0.71	0.92	0.001
Gender female	2.43	0.86	6.88	0.096
Age (year)	1.03	0.99	1.06	0.158
APACHE II score	0.99	0.93	1.05	0.618
Time to ROSC (min)	1.03	0.99	1.07	0.172
Bystander CPR	0.93	0.37	2.33	0.878
Ventricular fibrillation	0.55	0.21	1.44	0.223

**Pseudo R-squared: 0.557**				

CPR, cardiopulmonary resuscitation; ROSC, return of spontaneous circulation; CI, confidence interval; min, minutes.

### ROC analysis of outcome

**Figure 4:**
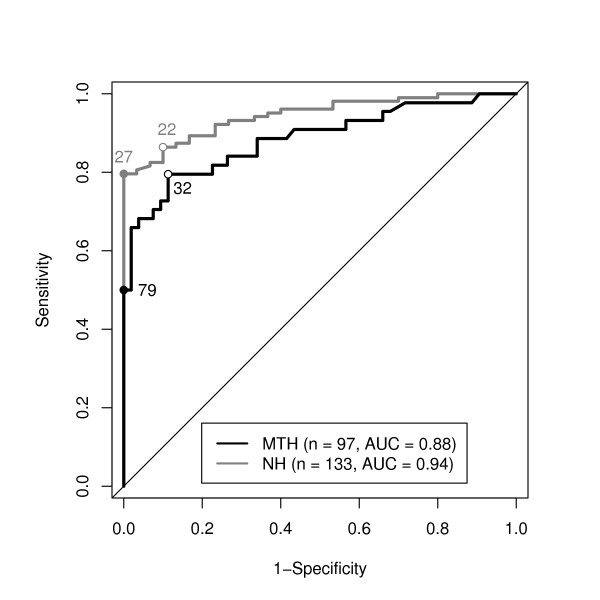
**ROC analysis of CPC 1 to 2 vs. CPC 3 to 5 for patients treated with mild therapeutic hypothermia and non-hypothermia group**. Circles with white background mark the best cutoff point (minimal distance to left upper corner); NSE cutoff values are given in μg/l. Solid circles mark cutoff points with 100% specificity.

ROC analysis for the prediction of a poor neurological outcome (CPC 3 to 5, Figure 4) showed a higher area under curve (AUC) in the non-hypothermia group (AUC 0.94) compared to the hypothermia group (AUC 0.88). The best NSE cutoff value (shortest distance to left upper corner) was higher under hypothermia treatment (31.8 μg/l; sensitivity 79.5%; specificity 88.7%) compared to the non-hypothermia group (22.4 μg/l; sensitivity 86.4%; specificity 90%). Cutoff values predicting unfavorable outcome (CPC 3 to 5) with a specificity of 100% were also higher in the hypothermia group (78.9 μg/l) compared to non-hypothermia (26.9 μg/l).

### Survival analysis

**Figure 5:**
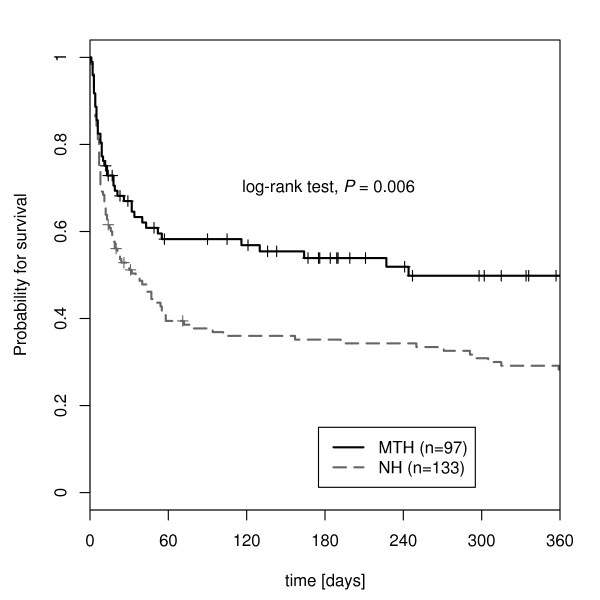
**Kaplan-Meier survival curves of hypothermia and non-hypothermia group**.

Kaplan-Meier analysis revealed a probability for 365-day survival of 45.9% (CI 33.3 to 57.7%) in the hypothermia group compared to 27.4% (CI 19.8 to 35.5%) in the non-hypothermia group. The log rank test was significant (*P *= 0.006; Figure 5).

## Discussion

Determination of serum NSE is an established tool for outcome prediction after cardiac arrest [5,9,10]. Nevertheless, post-resuscitation care has changed considerably with the introduction of therapeutic hypothermia in recent years. In general, outcome prediction may be more difficult in patients treated with hypothermia [11,12]. In particular, the role of NSE levels for outcome prediction in patients treated with MTH has only been investigated in a few studies with conflicting results [13,14]. Therefore, the predictive value of NSE in patients receiving MTH treatment requires re-evaluation.

The univariate analysis in the present study revealed a significantly lower median NSE serum level (72 hours after admission to hospital) under mild hypothermia compared to the non-hypothermia group. This result is in line with the published hypothesis of Tiainen *et al*. that the neuroprotective effect of hypothermia would be accompanied by diminished release of NSE [14]. Tiainen *et al*. analyzed the effect of therapeutic hypothermia on levels of serum NSE performing serial measurements at 24, 36 and 48 hours after cardiac arrest. In a repeated measurement analysis the levels of NSE were significant lower in patients treated with hypothermia compared to control group. However, the median of NSE at 48 hours (7.9 μg/l; IQR, 5.9 to 13.9 μg/l) in the hypothermia group was only slightly lower compared to the control group (median 8.6 μg/l; IQR, 5.2 to 20.2 μg/l). After adjusting our data for confounders, hypothermia treatment showed only a trend to lower NSE values without reaching significance. However, the low r-square of this model indicates that further unknown important factors may be relevant.

To analyze the association of NSE, neurological outcome and MTH treatment after cardiac arrest, we used a logistic regression model revealing that the neurological outcome was associated with hypothermia treatment and the interaction term of NSE and hypothermia treatment. This interaction demonstrates that the relation between NSE and neurological outcome is modified by hypothermia treatment (Figure 3). Thus, the effect of hypothermia on neurological outcome differs substantially in comparison to the non-hypothermia group, depending on serum NSE level at 72 hours. The greatest therapeutic effect of hypothermia was observed in patients with NSE levels between 20 and 80 μg/l whereas in patients with high (>80 μg/l) or low (<20 μg/l) NSE levels the difference to patients not treated with hypothermia proved to be limited. Therefore, MTH may not only result in lower NSE levels but it appears to be associated with the probability of a better neurological outcome in patients treated with MTH in comparison to NH patients with similar NSE levels.

The results of the ROC analysis are a further indicator that the relation between NSE and neurological outcome is substantially modified by hypothermia treatment as the non-hypothermia group revealed a higher area under curve (AUC, 0.94) compared to the hypothermia group (AUC, 0.88). This trend towards a reduced discriminatory power of serum NSE levels for poor neurological outcome under hypothermia treatment was also found by Tiainen *et al*. with an AUC of 0.89 in the control group compared to an AUC of 0.80 [14].

In our series, cutoff values predicting unfavorable outcome (CPC 3 to 5) with a specificity of 100% were higher in the MTH group (78.9 μg/l) compared to NH group (26.9 μg/l) resulting in a cutoff-ratio (MTH/NH) of 78.9/26.9 = 2.9. Tiainen *et al*. published a cutoff of 25.0 μg/l for NSE at 48 hours predicting six-month neurological outcome with a 100% specificity after MTH and 8.8 μg/l for the NH group (cutoff-ratio, 25.0/8.8 = 2.84) [14]. Although the level of NSE cutoff is lower compared to our study, the NSE-cutoff-ratios are similar. Oksanen *et al*. found a cutoff of 33 μg/l of NSE at 48 hours predicting poor six-month neurological outcome with a specificity of 100% whereas Rundgren *et al*. obtained a NSE cutoff of 27.7 μg/l at 48 hours and 27.3 μg/l at 72 hours [15,16].

The comparability of absolute NSE values for prediction of neurological outcome seems to be limited as cutoffs are presumably affected by multiple parameters including time to NSE measurement, laboratory immunoassay, cause of cardiac arrest, in-/out-of-hospital cardiac arrest, time to ROSC and outcome definition. Especially different methods of NSE analysis may interfere with a comparison of results from different laboratories. The present study assessed NSE values at 72 hours after admission to hospital. This time point was shown to be superior for prediction of neurological outcome in non-hypothermia cardiac arrest patients [5,9,10]. However, the ideal time point for measurement of NSE in patients treated with MTH for predicting outcome is still unclear. In a recent study, ROC analysis of neurological outcome showed an almost similar AUC (0.84) for NSE at 72 hours compared to NSE at 48 hours (AUC 0.83), whereas the threshold with 100% specificity of NSE at 48 hours was associated with a higher sensitivity (67%) compared to NSE at 72 hours (50%) [16]. Current published data suggest that the time course of NSE serum levels might be a better predictor for outcome than a single measurement of NSE.

This study also has some limitations that should be pointed out. Results were obtained from a single-centre trial using a retrospective sample of non-hypothermia patients rather than from a prospective randomized study. However, due to the fact that the current guidelines clearly recommend mild hypothermia treatment after cardiac arrest, a prospective randomized trial comparing hypothermia and non-hypothermia is ethically not acceptable. With our retrospective study design it cannot be excluded that along with the implementation of hypothermia treatment post-resuscitation care has developed and improved in general. Moreover, we did not collect data on neurological long-term outcome. However, it has been shown that CPC scores at hospital discharge may undergo only minor changes in the following six months [17-19].

A general problem in all studies on prognostication is the fact that these trials are susceptible to *self-fulfilling prophecies*. In our analysis, NSE levels were not blinded to the physicians in charge. Nevertheless, the process of decision making on therapy withdrawal in patients with poor neurological prognosis always consisted of a combination of clinical, electrophysiological and biochemical tests, which is in line with current practice [1,2,20]. As stated in the method section, both groups of patients received full ICU support over the first three days. Furthermore the length of ICU stay was shorter in the hypothermia group compared to the control group.

From the pathophysiological point of view our data are somewhat surprising. According to our results, patients treated with hypothermia stand a good chance of recovery despite higher levels of NSE. However, it cannot be fully ruled out that higher NSE levels in the hypothermia group may partly be due to factors other than hypoxic brain damage, for example a higher proportion of patients with non-convulsive status epilepticus. A current study by DeGiorgio *et al*. evaluated the NSE release in different subtypes of status epilepticus. The mean peak NSE level for non-convulsive status epilepticus was 37.83 ng/ml [21]. Moreover, the incidence of non-convulsive status epilepticus in survivors after cardiac arrest undergoing hypothermia amounts to 10% and appears to herald a fatal disease course in most patients [22]. Although we did not routinely perform electroencephalography in all patients, these data show that a higher incidence of status epilepticus does not sufficiently explain the differences in NSE levels between the groups.

Another possibility is that NSE serum levels reflect different mechanisms of neuronal damage that are differentially affected by MTH. Support for this hypothesis comes from investigation of stroke patients. Wunderlich *et al*. measured NSE on admission and on each of the first four days after stroke [23]. An early first NSE peak was interpreted as due to a rapid release out of the initially damaged tissue, followed by a second peak at 72 hours reflecting secondary mechanisms of brain damage, ongoing neuronal cell death or persistent disturbance of the blood brain barrier. NSE release in patients after resuscitation was found to peak after 72 hours in the majority of published studies including patients without hypothermia treatment. It remains to be demonstrated whether mild hypothermia treatment affects NSE release especially in the late phase after 48 to 72 hours where reperfusion injury, ongoing cell death, edema and dysfunction of the blood brain barrier take place. Therefore a different NSE kinetic in patients undergoing hypothermia is possible and the best time point for neurological outcome prediction with NSE is obviously still unknown in patients treated with MTH.

## Conclusions

In summary, in patients after cardiac arrest, single measurements of NSE should be interpreted with caution as many patient- and treatment-related factors may influence the amount and kinetics of NSE release. In accordance with our results, NSE levels drawn 72 hours after ROSC correlated poorly with neurological outcome according to current recommended cutoffs in patients treated with MTH. Therefore the usefulness of current NSE cutoff values for prognosis during post-resuscitation care seems limited in these patients. Decision on treatment continuation or discontinuation should therefore always be based on a full assessment of complimentary clinical observations and neurophysiological testing.

## Key messages

• The calculated interaction between NSE and hypothermia treatment was a significant outcome predictor.

• Survivors undergoing hypothermia treatment had remarkably higher NSE cutoff levels for bad outcome (CPC 3 to 5) compared to non-hypothermia patients.

• Currently recommended NSE cutoff levels after resuscitation do not reliably predict poor outcome.

• The probability for 365-day survival was significantly higher after hypothermia treatment.

## Abbreviations

AAN: American Academy of Neurology; AIC: Akaike information criterion; APACHE II: Acute Physiology and Chronic Health Evaluation II; AUC: area under the curve; CPC: cerebral performance category; CPR: cardiopulmonary resuscitation; ICU: intensive care unit; IQR: interquartile range; MICU: medical intensive care unit; MTH: mild therapeutic hypothermia; NH: non-hypothermia; NSE: neuron specific enolase; ROC: receiver operating curve; ROSC: return of spontaneous circulation.

## Competing interests

The authors declare that they have no competing interests.

## Authors' contributions

CS, IS, ED, and DH designed and supervised the analyses and analyzed all data. JS, FM and JN were involved in the collection of all data and participated in the data analyses. CP, AJ and AK participated in the design of the study, revised the manuscript for important intellectual content, and helped to draft the manuscript. DH and IS contributed equally to this work. All authors read and approved the final version of the manuscript.
